# Determinants of Unlawful File Sharing: A Scoping Review

**DOI:** 10.1371/journal.pone.0127921

**Published:** 2015-06-01

**Authors:** Steven James Watson, Daniel John Zizzo, Piers Fleming

**Affiliations:** 1 Department of Psychology, Lancaster University, Lancaster, Lancashire, United Kingdom; 2 BENC and Newcastle University Business School, Newcastle University, Newcastle Upon Tyne, Tyne and Wear, United Kingdom; 3 School of Psychology, University of East Anglia, Norwich, Norfolk, United Kingdom; Universidad Veracruzana, MEXICO

## Abstract

We employ a scoping review methodology to consider and assess the existing evidence on the determinants of unlawful file sharing (UFS) transparently and systematically. Based on the evidence, we build a simple conceptual framework to model the psychological decision to engage in UFS, purchase legally or do nothing. We identify social, moral, experiential, technical, legal and financial utility sources of the decision to purchase or to file share. They interact in complex ways. We consider the strength of evidence within these areas and note patterns of results. There is good evidence for influences on UFS within each of the identified determinants, particularly for self-reported measures, with more behavioral research needed. There are also indications that the reasons for UFS differ across media; more studies exploring media other than music are required.

## Introduction

This paper presents a simple conceptual framework to map out the existing available evidence regarding unlawful file sharing behavior (referred to as UFS hereafter) and models the decision to engage in the unauthorized consumption of copyright protected goods or to purchase those goods. We employ a *scoping review* that aims to be as systematic and transparent as possible, and which we borrow from areas such as public health and social policy research (e.g. [1, 2]). The application of a scoping review methodology to our context is novel within this field. We are also able to identify areas which require further empirical support in terms of the quantity and quality of available evidence.

The creative industries are worth £36.3 billion, and support 5% of employment and 270,000 businesses in the UK alone [3, 4]. However, these industries are purportedly at risk from unlawful distribution of creative works over the internet. The use of file sharing networks to acquire content for free is extremely common. One in six UK internet users consumes at least some content unlawfully online, or almost one in three of those that consume any content online [5]. Furthermore, peer-to-peer (p2p) file sharing networks are said to account for up to a third of all internet traffic [6]. Therefore, it is important to determine what research exists to explain why consumers choose to file share, or else choose to purchase when UFS is possible, and what are the limitations of that research. If we understand the mechanisms of user choice, this raises the possibility of developing strategies that can compete with UFS more effectively by targeting services to the needs of particular user groups [7].

Existing reviews on UFS are narrative summaries of the existing evidence. However, these are subject to various biases which do not allow them to be replicated or independently verified [8, 9]. The methods of study selection are not described and it is difficult for any one expert to remain up to date with the entire literature available on any one topic [10].

In order to investigate a broad research question, to determine the extent and nature of the research into the determinants of UFS and to provide an associated conceptual framework, the most appropriate method is a scoping review [11]. Scoping reviews specify the search strategy, inclusion and exclusion criteria, and principles of charting and coding data. The specific scope of the review as well as the inclusion criteria are refined iteratively during the data collection process as knowledge of the available evidence increases [1, 11]. It is not necessary to collect every available study on the topic. Instead, scoping reviews aim to cover the conceptual breadth of the available literature and identify the different types of evidence that have been put forward to answer relevant research questions [12]. The identified literature is then coded so that the variables and factors associated with UFS across a diverse array of literature can be meaningfully compared [1, 11, 13]. The net result of this process is a largely narrative account of the current state of play in a research area allowing for identification of research gaps and, potentially, the generation of theory for future empirical testing.

## Method

These results come from a scoping review on the determinants and implications of UFS of digital media consumed for entertainment, initially defined as music, film, television, electronic games, books and pornography. Our search strategy maximised the breadth of literature regarding UFS from English language academic and grey literature. “Grey literature” refers to any research not published in academic journals. For example government or industry reports, as well as academic research that has not been published.Keywords were developed that combined a range of methods of sharing with relevant types of content that could be shared. Additional keywords were excluded which introduced only irrelevant articles into the search. To ensure the search was comprehensive, identified articles were checked against those from the reference lists of previous literature reviews [14], specifically [15], [16] and [17]. The search string was refined until identified results indicated that the included articles were as comprehensive as possible, i.e. the search prioritizes sensitivity over specificity [12]. The search strategy is summarized in Table 1. To encompass a range of disciplines and perspectives the search string was utilized in five academic databases; Web of Knowledge, EconLit, Communication and Mass Media, PsychInfo, and LexisNexis.

**Table 1:** Search strategy for academic databases.

Modes of sharing:
(File sharing OR file-sharing OR DRM OR Digital rights manag* OR digital medi* OR File upload* OR File download* OR Torrent file* OR peer-to-peer OR peer to peer OR p2p OR usenet OR freenet OR Newsgroup OR File transfer protocol OR ftp OR shared directory OR Piracy OR pirat* OR online piracy OR copywrit* OR intellectual property OR forum OR digital economy OR kazaa OR Limewire OR bittorrent OR Pirate Bay OR Napster OR isohunt OR eDonkey OR gnutella OR megaupload)
AND: Content shared
(video game OR video-game OR game OR gamer OR gaming OR electronic games OR digital game* OR digital music OR Music OR iTunes OR Album OR sound record* OR Music record* OR artist OR record sales OR DVD sales OR music purchas* OR DVD purchas* OR DVD OR film upload* OR film download* OR movie upload*OR movie download* OR motion picture* OR ebook OR e-book OR e book OR digital book* OR TV OR television OR tele vision OR tele-vision OR tele OR pornography OR porn OR xxx OR adult entertainment OR adult movie OR creativ* OR creator OR artist* OR entertain* OR attitude* OR intention OR social norm*)
NOT: Noise inducing keywords
(Medical OR medicine OR medieval OR Navy OR naval OR maritime)

Note: * refers to the wildcard character in the academic databases that accounts for words that may have multiple endings. E.g. ‘Download*’ would return ‘download’, ‘downloading’ and ‘downloaded’ *etc*.

Due to search string incompatibility a reduced search was performed in the Westlaw database. It was “(piracy OR file sharing) AND (music OR books OR video games OR film OR television OR pornography)”. To capture pre-publication articles, the database of working papers “Social Science Research Network” was searched for the past four full years (2009–2013) using the keywords “file sharing” and “piracy”. This database does not support Boolean operators and the two searches were run separately and manually combined. Searches were performed and articles extracted from the 20^th^ to 27^th^ of February 2013.

Grey literature was sought on the websites of key stakeholders and research centers that investigate UFS. Where necessary, the organizations were contacted and access to any research requested. Table 2 lists organizations from which grey literature was sought.

**Table 2:** Attempted sources of grey literature.

Organizations from which literature was sought
Intellectual Property Office (IPO)
Ofcom
The European Commission
Federal Trade Commission (FTC)
Consumer Focus
Organisation for Economic Co-operation and Development (OECD)
Performing Right Society for Music (PRS)
International Federation of the Phonographic Industry (IFPI)
UK Music
Federation Against Copyright Theft (FACT)
Creative Coalition Campaign (CCC)
Alliance for Intellectual Property
British Phonographic Industry (BPI)
Association for United Kingdom Interactive Entertainment (UKIE)
Institute for Information Law (IVIR)

### Relevance Screening

One author (SJW) screened the titles of all identified articles. After excluding obviously irrelevant articles, two authors (SJW and PF) independently screened a random sample of 100 abstracts for inclusion. The decision to include the selected articles was discussed between the two authors to refine inclusion and exclusion criteria and the scope of the review. This promotes consistency in screening and limits single author bias. All remaining abstracts were screened by one author (SJW). Articles were retained for full text review where the abstract indicated that inclusion criteria may be met. Full text review was conducted by one author (SJW).

### Inclusion and Exclusion Criteria and Data Extraction

Scoping reviews develop inclusion and exclusion criteria iteratively during the process of screening articles [1]. It became clear that it would be necessary to include unlawful acquisition of software not intended for entertainment use as well as entertainment media to fully explore motivations for UFS. Similarly, for practical reasons, the scope of the review was narrowed to cover the last 10 years of UFS research in the period between January 2003 and February 2013.

This analysis and the identified research focus upon individuals choosing to download copyrighted materials, though we recognize the growing importance of streaming. Only unlawful sharing of otherwise legal content is considered, i.e. studies exploring materials such as child pornography were excluded. Similarly, we focus upon the informal transfer of files between peers without financial transaction.

Only empirical studies were included. The included studies were limited to those including human participants, i.e. not theoretical models without primary observation. Only reports published in the English language are included. Table 3 has a summary of inclusion and exclusion criteria.

**Table 3:** Summary of inclusion and exclusion criteria.

Inclusion Criteria
*To be included research must*:
Explore the causes or consequences of UFS of digital media—Digital media is restricted to: Music, movies, software, TV shows, videogames, e-books, and pornography
Be published after January 1^st^ 2003 inclusive
Be published in the English language
Exclusion criteria
*Research is excluded where*:
Media files are acquired via a financial transaction
Media files contain illegal material (e.g. child pornography)
No novel data is presented (e.g. reviews, opinion pieces, dual publications)
No empirical testing on human participants is performed (e.g. pure economic models)
The article is not written in the English language

### Outcome Measure

Data was extracted and categorized iteratively. The type of evidence was characterized in terms of the *distance of the unit of measurement from actual behavior*, which is what ultimately we are interested in when considering UFS and its welfare implications. Most distant from actual behavior, we have *stated preferences and attitudes* on how good or bad, right or wrong, an action is perceived to be, and *stated intentions to perform behavior*, i.e. to engage in UFS behavior. Closer to actual behavior are *willingness to pay (WTP)* measuring the amount of money that people state they are willing to pay to obtain a good and *stated behavior*, which is a participant’s report of behavior that has occurred in the past, typically as stated in a survey. We classify a study as looking at *observed behavior* if it is behavior directly observed either at an individual or population level: behavioral experimental data and sales data fit into this category. Qualitative data is considered somewhat separate from this hierarchy as this can examine reports of stated behavior and preferences in a holistic manner. Table 4 summarizes the hierarchy of outcome measures.

**Table 4:** Definition of outcome measures for unlawful file sharing.

Outcome Measure	Definition
*Qualitative research*	Explorations of perceptions of or engagement in behaviors without quantitative assessment.
*Stated preferences and attitudes*	Outcome is at the level of how good or bad, right or wrong, or preferable an action is perceived to be
*Intentions to perform behavior*	Outcome described participants reports of behavior that they plan to engage in in the future
*Willingness to pay (WTP)*	Outcome represents the amount of money that a participant states they are willing to pay in order to obtain a good
*Stated behavior*	Outcome represents a participant’s report of behavior that has been engaged in in the past, such as from a survey
*Observed behavior*	Outcome represents behavior that is either directly observed at the level of the individual, such as in an experiment, or else at the population level, such as from sales data

### Data Analysis

Data analysis was based upon thematic framework analysis [13, 18, 19]. Data were initially coded during extraction according to relatively *ad hoc* groups of similar variables. These were developed and refined into a framework in which the proposed correlates of UFS could be incorporated. The results within each subtheme were divided according to level of outcome measurement, and medium type (music, movies, TV, videogames, software, books or pornography). This allows comparison of the relative impacts of variables across different measures of outcome and media. A summary of the key points from each theme is presented. The supplements contain the full list of references (S1 File) and the data extraction form (S2 File).

### Selection of Studies

**Fig 1:**
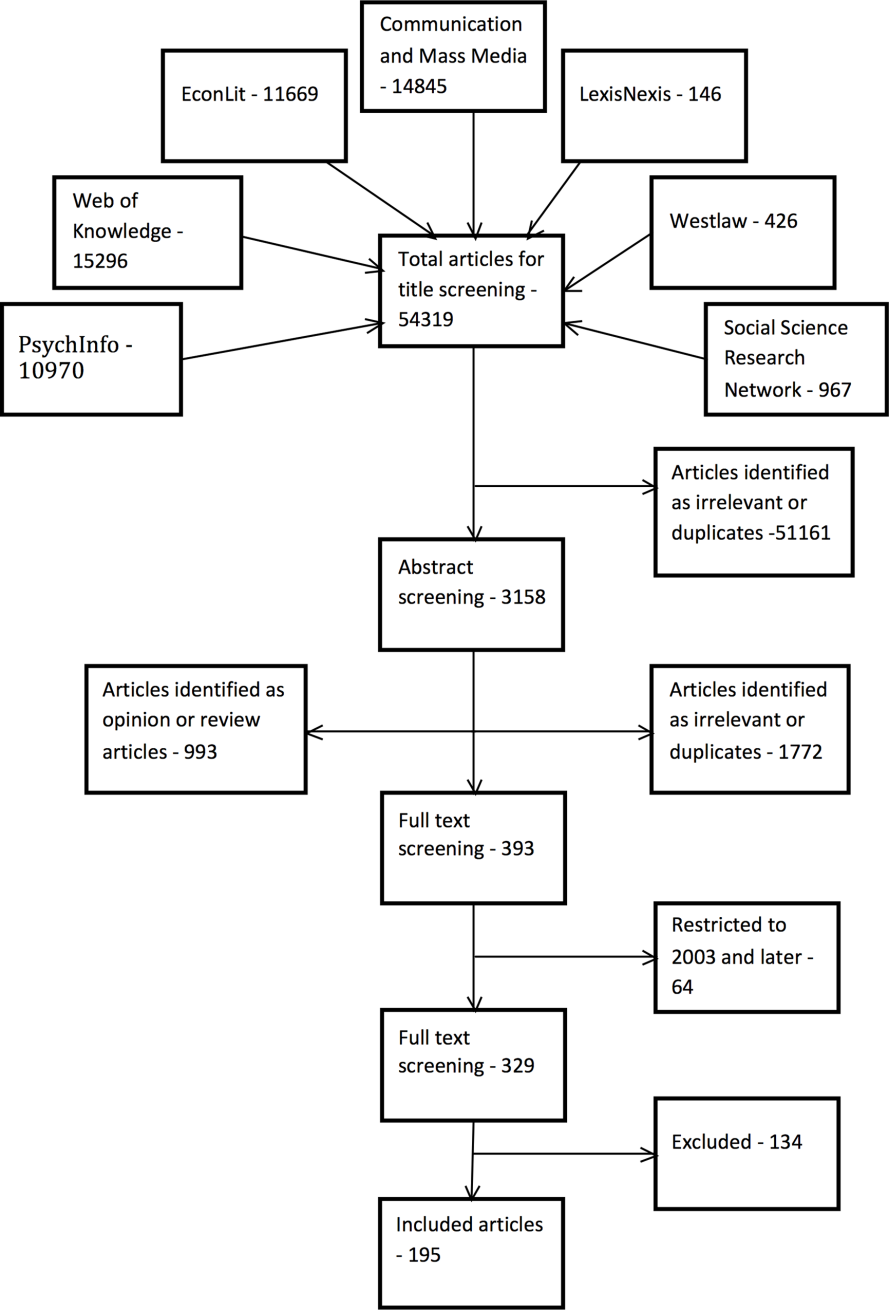
Flow diagram of academic articles included in the review (in addition to 14 grey literature articles).

A large majority of the initially identified studies were excluded, this is common when searches are highly sensitive [12]. Fig 1 summarizes the inclusion and exclusion process for all articles identified through electronic databases. Reasons for excluding 134 articles at full text review were, not being an empirical study (46), not being relevant (43), being a duplicate publication (37), only examining exchanges which included a financial transfer (7), and one study was excluded for being in a foreign language. This left 195 articles to be included in the review, though only 192 were useable. Three studies did not provide data that could be synthesized into any of the categories of our conceptual framework and so were not used further. They only compared UFS attitudes and behavior depending upon occupation [20], provided a typology of those that UFS but without presenting sufficient information for the individual factors that determined this typology to be extracted and combined with similar studies [21], or else provided insufficient description of the variables included in their model to permit accurate classification of included factors [22]. One hundred and twenty-two reports were identified from the grey literature, of which 108 were excluded. The reasons for exclusion were not being empirical (48), not being relevant (43), being a duplicate publication of an already identified article (13), only examining exchanges of media which included a financial transfer (2), and being published in a foreign language (2). This included an additional 14 articles in the review. Therefore the final number of studies included in the review is 192 + 14 = 206. Only one article made any reference to pornography as a media [23], and in this case no predictors of unlawful pornography downloading were identified. Therefore this media was not analyzed further.

## Results

In considering the evidence, it became clear that we could classify it depending on the type of factors affecting whether or not to engage in unlawful file sharing. To anticipate the conceptual framework presented later in the paper, we can consider legal and financial sources, experiential sources, technical sources, social sources and moral sources of utility. What we are going to label a *total net utility* judgment, i.e. one that takes into account (possibly in a interacting and non-linear way, as will be shown) all utility sources, then determines whether or not to engage in UFS, buy the product or do nothing, depending on which action leads the highest utility.

We can classify data from 186 of the 206 included studies from our scoping review according to the net utility source. Some studies (also) refer to total net utility estimates regarding how beneficial or otherwise UFS is perceived to be when compared to the options of legal purchase or no action; they are classified as providing information on total net utility.

**Fig 2:**
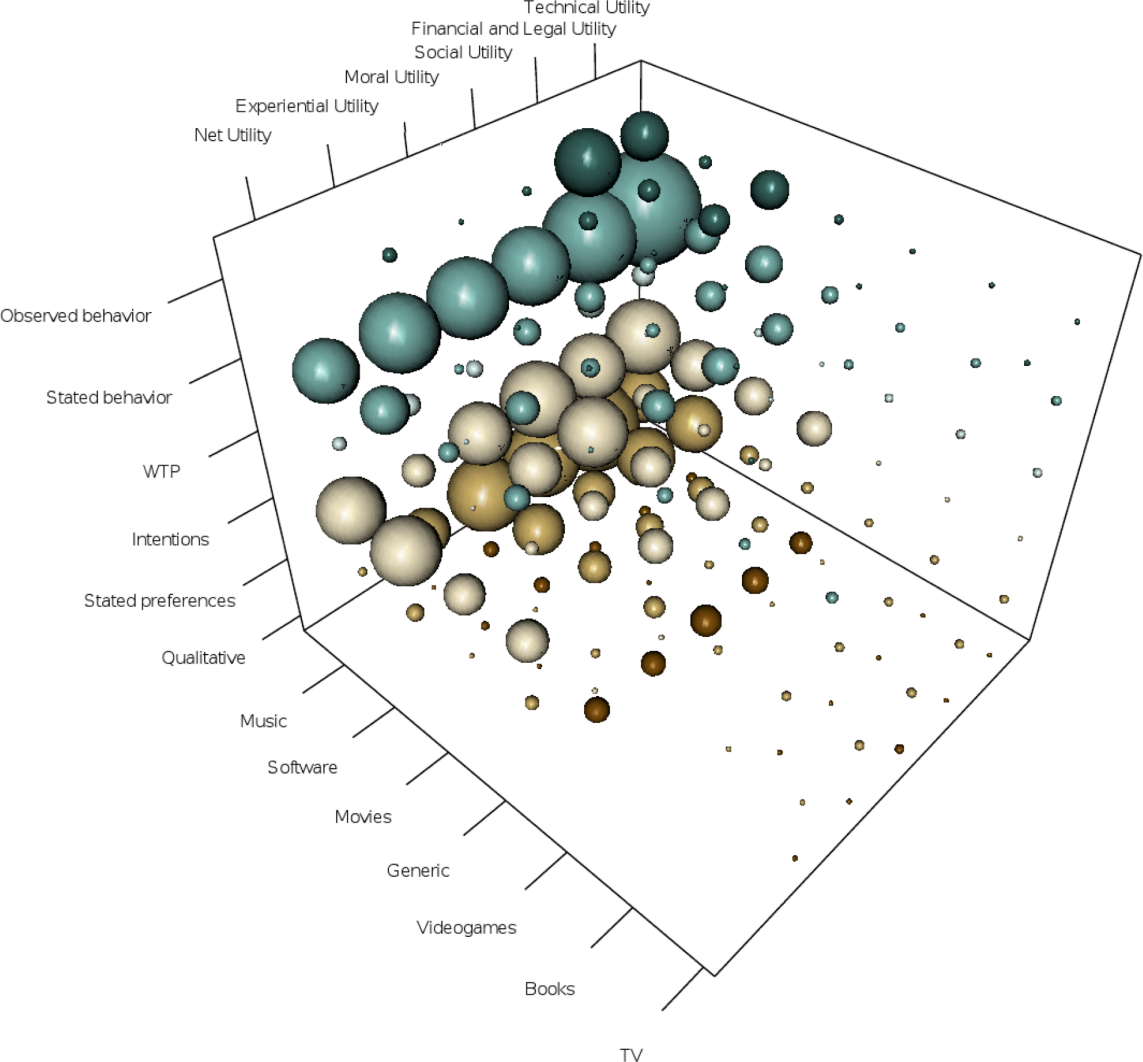
A Cubic Space of Available Evidence on Unlawful File Sharing. Note: the size of the sphere in the cube represents the amount of evidence available for each combination.

We provide a conceptual representation of the available literature along three dimensions: (1) the net utility source or total net utility, as discussed; (2) the market medium (music, software, movies, TV, books and videogames); and (3) the outcome measure (qualitative, stated preferences, intentions, willingness to pay WTP, stated behavior, observed behavior). We illustrate this in Fig 2 and in Table 5. Note that Individual studies may be counted in multiple cells, e.g. a single study may cover both music and movies.

**Table 5:** Number of observations for each utility level, market medium and outcome measure.

Utility	Medium	Qualitative	Stated preferences	Intentions	WTP	Stated behavior	Observed behavior	Total
Financial and Legal Utility	Videogames	0	1	0	0	2	1	4
	Books	1	2	0	0	0	0	3
	TV	1	2	0	0	0	1	4
	Movies	0	7	3	0	7	7	24
	Software	3	16	7	1	4	5	36
	Music	3	26	18	7	24	16	94
	Generic	7	4	3	1	7	0	22
	Total	15	58	31	9	44	30	187
Experiential Utility	Videogames	0	0	1	0	3	0	4
	Books	0	1	0	0	2	1	4
	TV	1	1	0	0	2	0	4
	Movies	1	1	3	0	7	1	13
	Software	2	0	1	1	2	0	6
	Music	1	12	8	5	18	3	47
	Generic	6	2	0	0	1	1	10
	Total	11	17	13	6	35	6	88
Technical Utility	Videogames	0	2	1	2	2	1	8
	Books	1	2	1	2	2	1	9
	TV	1	2	1	2	2	1	9
	Movies	0	5	10	2	9	9	35
	Software	3	16	14	1	9	3	46
	Music	1	16	21	6	30	12	86
	Generic	6	3	9	1	4	2	25
	Total	12	46	57	16	58	29	218
Social Utility	Videogames	0	1	0	0	0	0	1
	Books	1	2	0	0	0	0	3
	TV	2	2	0	0	0	0	4
	Movies	1	7	10	0	3	1	22
	Software	3	11	18	1	7	4	44
	Music	4	21	20	1	19	2	67
	Generic	8	2	8	0	8	1	27
	Total	19	46	56	2	37	8	168
Moral Utility	Videogames	0	2	0	0	0	1	3
	Books	1	2	0	0	0	0	3
	TV	1	2	0	0	0	0	3
	Movies	0	8	7	0	4	0	19
	Software	4	13	13	0	6	0	36
	Music	4	20	16	4	19	1	64
	Generic	6	5	8	0	7	0	26
	Total	16	52	44	4	36	2	154
Total Net Utility	Videogames	0	0	1	0	0	0	1
	Books	0	0	0	0	0	0	0
	TV	0	0	0	0	0	0	0
	Movies	0	1	9	0	4	0	14
	Software	0	4	16	0	10	0	30
	Music	0	2	16	3	14	0	35
	Generic	0	3	9	0	5	0	17
	Total	0	10	51	3	33	0	97

It is clear from Fig 2 and Table 5 that books, videogames, and TV have received very little attention. There is some literature represented that explores software and movie UFS, but by far the most common medium explored is music. A number also ask participants to report on “digital piracy”, “p2p use” or “downloading of digital media” generally. The reliance on a generic description of behavior seems likely to generate measurement error. To prove an association between a proposed cause of a behavior and the behavior itself, it is important to be specific about the target behavior. Motives to download music unlawfully may differ from motives to download movies or software; this review finds potentially different financial and experiential effects for software compared to other media (see below). Asking participants for reasons they download media in general, leaves participants and researchers unable to specify which behaviors are being considered and thus introduces noise into estimates of association. Furthermore, estimates of behavior using actual observation are rare when compared to outcomes based upon perceptions of UFS, stated intentions to file share or purchase, or stated behaviors. Almost all of the studies of observed behavior concern financial and legal net utility and technical net utility, and even then are mostly restricted to music and movies. Most observations look at attitudes (stated preferences) and intentions, and it is unclear whether these would result in actual behavior, or where there is a relationship, whether they may reflect reverse causality (from behavior to statements made congruent to the behavior).

We now consider the existing evidence with respect to total net utility and each net utility source in detail.

### Total Net Utility

Using global attitude assessments or reported cost-benefit assessments as a proxy for a net utility assessment provided evidence that these measures reflect consumer preferences regarding UFS.

There is overwhelming evidence for a relationship between *attitudes regarding UFS and intentions to file share* or purchase; only one study from 32 failed to find a relationship between the two variables [24]. Furthermore, all nine studies which estimated the *impact of attitudes upon stated UFS behavior* identified a relationship regardless of medium. One study explored the relationship between attitudes and legal purchases, and failed to identify a relationship between attitudes regarding UFS and legal purchases of digital music [25]. Intent to file share or purchase was similarly found to be associated with stated purchasing and UFS behaviors regardless of medium.

Hsu and Shiue [26] found participants with a higher WTP for software preferred legal over unlawful software, had higher legal purchase intentions, and were more likely to report having purchased legal software. There is also evidence from a further nine studies that those that engage in UFS have a lower WTP for content than those that do not, and that unlawfully downloaded content was valued less than purchased content [27]. It is clear overall that *WTP is related to UFS*.

The evidence base for net utility assessments comprised 97 outcomes, skewed towards music (36%), and software (31%). About half as many were relevant to movie UFS (14%), and only one was relevant to videogames. No studies utilized net utility estimates to investigate e-book or TV series UFS. 61 (63%) of the available outcome estimates utilized stated preferences or intentions as an outcome measure. While 33 outcomes (34%) referred to estimates of stated behavior, no study utilized observed behavior for total net utility.

### Legal and Financial Utility

Nine studies compared the intellectual property (IP) *law strength* in different nations to estimate rates of UFS or legal sales. Overall, indicators of legal strength such as membership of international treaties, and legal enforcement costs and efficiency were associated with more legal sales and lower rates of UFS. The studies utilized estimates of UFS exclusively from the producers’ side of the IP rights debates, such as the International Federation of the Phonographic Industry, Business Software Alliance, and the International Intellectual Property Alliance. A more fundamental problem is that correlation is not causation. For example, different cultural factors or economic development levels may be correlated with particular IP regimes and with particular levels of UFS. Relatedly, IP law strength was also correlated with national wealth which may confound the extent to which the two effects can be disentangled; although Walls [28] model implies that for unlawful movie file sharing legal strength may be the more important factor. The role of national income may not be linear, with some evidence that when income is high sales of “inferior goods” such as music may drop [29]. For software, four studies consistently found that higher national income was associated with less UFS, suggesting that conversely software is not an inferior good.

There is evidence for a *temporary effect of new laws* protecting IP. Adermon and Liang [30] monitored internet traffic after a new IP law was introduced in Sweden, and compared this to Norway and Finland where no new law was introduced. Internet traffic immediately after the introduction of the new law reduced by 18%, but had recovered within 6 months of the law being passed, implying the new law acted only as a short term deterrent. Blackburn [31] similarly found that, when the Recording Industry Association of America (RIAA) announced lawsuits against individual file sharers, the availability of files on five torrent sites dropped. However, by the time lawsuits were actually being filed, overall availability of files had actually increased. Danaher *et al*. [32] (This paper has since been accepted for publication in the Journal of Industrial Economics after peer review) found that iTunes sales increased when the French public became widely aware of the incoming HADOPI law, which suggests that legal disutilities from unlawful sharing had increased legal sales. However, when the law was actually passed, no effect was observed. This may be because the French public had already fully adjusted to the policy change. Bhattacharjee *et al*. [33] found that the RIAA lawsuits reduced the number of files available for UFS, but a significant proportion remained available.

An alternative approach is to examine the impact of deterrents upon individual attitudes and behavior. Three groups of variables were considered: those that examined the *legal risk* (that is, the probability of capture), those that examined the severity of the consequences of capture, and those that did not distinguish between the two effects (22 studies). Of the latter 22 studies, eight estimated the impact upon actual behavior, four of which found deterrents reduced behavior, and four did not. Evidence was more convincing that a legal deterrent lowers intent to UFS, with four of five studies finding an effect.

Participants also stated that they did perceive laws to be a deterrent against UFS [34], but in most cases *perception of laws* as a deterrent was seen as a concern to a minority and more participants were concerned by technical risks of UFS such as catching computer viruses [35, 36]. Qualitative research also emphasized that participants thought that UFS “did not feel like a crime” [37], and that participants were often unaware of what was or was not unlawful [38].

Studies on the role of probability and severity of consequences specifically, found conflicting evidence. Two out of six studies examining the role of severity upon stated behaviors found a relationship, but four out of five studies on attitudes found a relationship. Conversely, evidence linking attitudes to the probability of capture were less clearly related (three of seven studies found a relationship) compared to behavioral evidence (five of seven studies find a relationship).

Fifty-three studies examined the role of pricing. With regard to behavior, higher prices were related to lower legal sales, though the link to UFS rates was unclear, with only five of 11 studies finding that higher prices were associated with more UFS. Six studies found a relationship between higher prices and lower observed sales of music [39–41] and movies [42, 43] and TV [44], no studies identified a relationship between higher pricing and observed UFS behavior. There was also no clear effect from 27 studies investigating the role of individual participant’s income upon UFS.

Legal and financial net utility has been heavily researched in comparison to the other identified utility types with 187 observations. However, there was still significant skew in the type of evidence available as well as the media represented. Half of observations focused on music UFS and purchasing. The evidence for other media types was much smaller at 19% of observations for software and 13% for movies. Only four studies explored TV and videogames, and three investigated e-books. Stated preferences and intentions were common outcome measures (31% and 17% respectively). 16% used observed behavior, and 24% had stated behavior as an outcome.

### Experiential Utility

Twenty studies explored *interest in the product* as an experiential factor, though only one used observed behavior [45]. Qualitative research from five studies indicated that enjoying the product was a stated motive for UFS in all media, with the possible exception of software. Konstantakis *et al*. [46] found that computer science students considered software to just be a tool they utilized and there was no emotional engagement with products employing this medium when compared to that with books, games, or other media. Five studies examined the role of interest in the product upon unlawful acquisition and only two found a significant positive association [47, 48].

Only four studies investigated *collecting* (the desire to own a collection) as a motive for UFS. Two studies found that it was associated with the gratification felt from unlawfully obtaining content [49, 50]. However, [51] found that 15% of her sample reported the desire to own a collection was a reason for preferring CDs over p2p files. Hennig-Thurau *et al*. [52] estimated the impact of desiring a large collection on actual behavior, and found that this was associated with downloading more movies unlawfully but not with the number of unlawfully acquired movies actually watched. Of course, having a large collection because of other reasons may have led to post hoc justifications such as stating a desire to own a collection.

13 studies examined the extent to which *sampling* (the desire to gain knowledge about product content) was a motive for UFS. These studies found broad support for the hypothesis that products were acquired unlawfully at least sometimes to try out content prior to purchase. Chen *et al*. [53] found that a reason for preferring the use of p2p services over legal content providers was that the p2p services were considered superior for trying new music (see also, [54]). UFS may also be utilized to obtain *niche content*, i.e. content which is not popular enough to be readily available legally. This possibility was explored in 16 studies. Six qualitative studies and three national surveys found support for this hypothesis, with participants stating that the availability of niche content on p2p services allowed access to content that would be otherwise unavailable. Tzantzara and Economides [55] had participants rank services in terms of the extent of their catalogue and found that p2p services were rated most highly while iTunes, the legal service examined, was ranked as the worst. Two studies provided empirical support that the belief that unlawful networks had the best selection of content was associated with using these services [56, 57]. Experential utility related to niche content may of course interact with technical utility from the technical availability of the content itself, which is discussed below.

Mateus and Peha [23] tracked the files that were downloaded via p2p networks on a university campus. They were able to demonstrate that content did follow a long tail distribution, implying that much downloaded content was not exceptionally popular.

The experiential utility evidence base was the slimmest, with only 88 outcome estimates. This was again heavily skewed towards music (53%) with other evidence limited; movies were the next most investigated media (15%). Most studies measured the impact of experiential factors on the level of stated behavior (40%), only six estimates (7%) utilized observed behavior.

### Technical Utility

The question of the *technical availability* of legal (particularly niche) content was explored by nine studies, five of which were qualitative, a survey, and three which estimated observed behavior. The qualitative studies emphasized the impact of release lags, also identified as a motive for UFS in a large survey of UK residents [35]. The impact of release lags received quantitative support from Danaher and Waldfogel [58], who found that longer release lags between countries for movies were associated with lower box office earnings in cinemas. Danaher *et al*. [44] found that the removal of NBC content from iTunes did not affect physical DVD sales but did increase the amount of UFS activity regarding NBC content on p2p networks. This implies that the physical and digital markets may be separate, with legal and unlawful downloads competing more closely than physical sales. Danaher *et al*. [44]Danaher, Dhanasobhon (44) also found that uploads and downloads of non-NBC content on p2p networks increased following the removal of the legal content. This could mean that, once some users had turned to UFS, they used this medium for more than just the removed content, suggesting that there are fixed costs associated with learning to engage in UFS and, once these are taken up, the technical disutility from engaging in UFS will be lower, leading to increased UFS. In Danaher *et al*. (2010), the restoration of NBC content to iTunes did not significantly reduce UFS activity, indicating that the removal of content had led to UFS becoming habitual.

Thirty-five studies have explored directly the hypothesis that, the easier UFS is, the more consumers engage in it. They have predominantly asked participants about internet access, or estimated it by measuring internet and broadband penetration. The evidence does not support an impact of these variables upon attitudes about UFS or intent to engage in UFS. Similarly the effect upon stated behavior in 12 studies was mixed, with six studies finding that technical ease of UFS was associated with UFS rates or a reduction in sales, four studies finding no effect, and two studies finding mixed effects. Mandel and Suessmuth [59] found that having a broadband connection was associated with engaging in UFS more frequently but not to a greater extent than those without a broadband connection. With regard to observed behavior, estimates of the impact of internet or broadband penetration were that it lowers physical music sales in five of six studies, though of course this can be interpreted in terms of online sales, not necessarily in terms of UFS. The one study with no effect, did find that there may be a relationship when IP law strength is weaker in a nation [39]. The direction of effect between broadband or internet penetration and sales was less clear for software, where a positive association between penetration and sales was found [60], and for movies, where two out of three studies found a positive effect upon sales [42, 43, 61]. A particular complication of using broadband or internet penetration as a measure of technical ease of UFS is that it is confounded by other variables such as legal online sales, wealth, legal strength, and national infrastructure. Similarly, there are difficulties with establishing a causal relationship between the availability of internet or broadband connections and UFS.

An alternative approach is to estimate the impact of the availability of UFS technology or websites upon sales. Danaher and Waldfogel [58] found that, after the introduction of the BitTorrent protocol, longer release windows were associated with lower box office revenues. This implied that pre-release movie UFS significantly impacted upon sales once UFS became easier to perform. Danaher and Smith [62] explored the impact of the shutdown of a major file sharing website (this paper has since been accepted for publication in the International Journal of Industrial Organization after peer review). They checked for differences in the use of this website between countries and identified a statistically significant increase in digital movie sales. However, it is impossible to determine from this study whether the observed increase would last beyond the 18 week follow up period. Poort and Leenheer [63] found that the blocking of the Pirate Bay website had led to 21% of participants reporting less UFS, but had had no effect on 72%, while 5% said they downloaded more. Of course, the effects may be moderated by the availability of substitute websites.

The evidence from 33 studies overwhelmingly indicates a relationship between perceived *technical skills* to engage in and having more positive attitudes towards UFS, and having a greater intent to file share. However only six studies estimated the impact of such perceptions upon stated behaviors. Although four of these six studies did find a positive relationship, there is a lack of evidence based upon observed behavior. Estimates of the impact of technical skill upon UFS were estimated by 31 studies. The evidence was reasonably consistent that greater technical skill was associated with a greater propensity to UFS behavior, however this was not found for three studies specifically examining software UFS [64–66].

One function of technical skill may be mitigating the *technical risk* posed by viruses and malware, which was explored by 19 studies. Qualitative studies and surveys including participants that did not file share found technical risks to be a disincentive for UFS. However, within samples of file sharers, technical risks were not consistently related to intent to file share or stated behavior. This may reflect that the perception of technical risks are more significant than the actual barriers themselves which once overcome no longer have any effect upon behavior [67]; alternatively, it may be related to the gap between intentions and actual behavior.

Seven studies suggested that one motive for UFS was having an *interest in technology*. Users who file share were found to be early adopters of technology [51, 68, 69] and qualitative evidence suggested that some file sharers do so to “push the envelope” [38]. Relatedly, UFS was perceived as being more convenient in terms of time taken to identify and acquire media and more flexible with regard to how files could be manipulated and used once acquired [51, 57, 70–73]. The use of digital rights management (DRM) and the requirement to continually relicense software products was opposed by users [74].

Technical factors associated with UFS have been relatively extensively researched, with 218 observations. Although music was still the most explored media type (39%), software (21%) and movies (16%) also had a number of relevant observations. However fewer than 10 observations each explored the influence of technical factors upon UFS and purchases of TV, videogames, and books. Stated behaviors, intentions and preferences were all explored to a similar extent (27%, 26% and 21% of outcomes respectively).

### Social Utility

A large number of observations (78) investigated *normative beliefs*, that is the extent the beliefs and behaviors of others could influence perceptions of UFS and UFS behaviors. There was widespread evidence that the perceived beliefs and behaviors of others were correlated to individual’s beliefs, intentions, and stated behaviors regarding engagement in UFS. One specific channel could be by moderating the effect of perceived legal strength. Of course, it may be that behavior—determined by other utility sources—may be determining responses in these studies. According to these studies, social norms may be more influential when norms are congruent with attitudes [75], when media is intended to be enjoyed socially rather than alone [76] and in collectivist versus individualist cultures [77]. The role of collectivist cultures upon UFS was separately analyzed in nine studies and within them UFS was perceived more acceptable and engaged in more frequently. Denegri-Knott [78] analyzed comments on a website that was set up in opposition to the RIAA and found that a number of them made reference to the fact that the internet itself may have a collectivist culture. Social norms may in part reflect wider cultural moral beliefs and we focus on moral utility below.

An additional social motive identified for engaging in UFS was maintenance of *social prestige*. Maintaining or enhancing reputation through knowledge of media was identified as a motive for UFS in nine qualitative studies. That said, three studies found a relationship between social-status seeking and UFS and three did not. Kwan [79] found that the relationship between the expectation of social gain from UFS and intent to file share was stronger for hedonistic media, movies and music, than for software. Social prestige may also be more important for providing content than for acquiring it [80, 81], but it is very difficult to know from this literature whether and how actual behavior would change as a result of changes in this potential source of social net utility. Social prestige may interact with experiential utility from the collecting motive, which we have discussed above.

A final social factor that may be related to UFS and legal purchases is *reciprocation*, that is the formation of reciprocal relationships. Qualitative studies identified that within file sharing networks it was felt that downloaders should contribute back into the system, and some networks enforce this by slowing download speeds for those that don’t contribute or barring non-contributory users entirely [73, 82]. Cenite *et al*. [70] found via interviews with students that there was also a sense of reciprocity with creators and that content that is enjoyed should be purchased. However, no studies have so far estimated the impact of reciprocity upon downloading behavior.

Most of the 168 sources of social net utility investigated music (40%) or software (26%). Movie UFS was the next most studied media (13%). TV series, books and videogames were investigated in less than five samples each. The most popular outcome measure was at the level of intentions (33%), followed by stated preferences (27%) and stated behaviors (22%). Only eight outcomes measured observed behaviors (5%).

### Moral Utility

Six studies directly compared the effects of participants basing their moral beliefs upon what was legal or not. Those participants that used the law to frame their own moral beliefs were more likely to perceive UFS as morally wrong, while, in a Swedish sample that considered UFS morally acceptable, the correctness of the laws was questioned [83].

Twelve studies examined the role different *moral frameworks* may have upon UFS. The contrast between absolutist versus relativist morals may have an impact upon both attitudes about and intention to engage in UFS [84] as well as reported behaviors [85] with absolutist moral frameworks being more likely to view UFS negatively. Research examining whether or not participants consider UFS to be a moral issue at all also found a division between those that utilized more absolute stances and those that instead used contextual information to inform their moral beliefs. Zamoon and Curley [86] performed a content analysis of the five most popular US newspapers, with articles on unlawful software file sharing coded according to their moral arguments for or against UFS. They found that arguments in favor of UFS were less likely than arguments against UFS to be based upon moral justifications. Arguments against UFS were centered upon a small number of key concerns, in particular economic harms to producers. Jambon and Smetana [48] asked participants to complete a survey when considering a scenario in which an artist receives 90% of the profits and a scenario in which industry receives 90% of the profits from sales. They found that it was argued that UFS was a personal and not a moral choice more often when artists were perceived to be the primary beneficiary of sales. It was proposed that, while arguments against UFS tend to focus on absolutes and are relatively constant, the arguments in favor of UFS are more fluid and shift depending upon context. Not considering UFS to be an ethical problem was found to be associated with UFS behaviors in both qualitative and quantitative studies [46, 87, 88].

37 studies tried to estimate the relationships between moral beliefs about UFS, and various *moral justifications* regarding UFS and behavior. Appealing to higher loyalties was a technique utilized more often by those that engaged in UFS more frequently indicating that a belief that UFS enhances social welfare may motivate such behavior [89]. Nine studies directly estimated this possibility and the evidence was consistent that the belief UFS enhanced social welfare was associated with attitudes, intentions, and stated behaviors regarding UFS. A further 15 studies investigated the role of participants awareness of causing harm via UFS and there was a general perception that little or no harm was caused, and while participants had more sympathy when harm was presented as being caused to creators rather than industry, there was still a perception that such harm could be absorbed easily.

Moral net utility was examined by 154 observations. Music was still the most common medium investigated (42%). Software was also fairly well represented in this literature (23%), with movies being researched to a lesser extent (12%). However, it was common to not specify media types when exploring moral factors (17%). As with other utility sources, videogames, TV series and books were only weakly represented. Measured outcomes were most commonly stated preferences, intentions and stated behaviors (34%, 29% and 23% respectively), but there was a paucity of evidence estimating observed behavior, with only two observations (1%).

## Discussion

**Fig 3:**
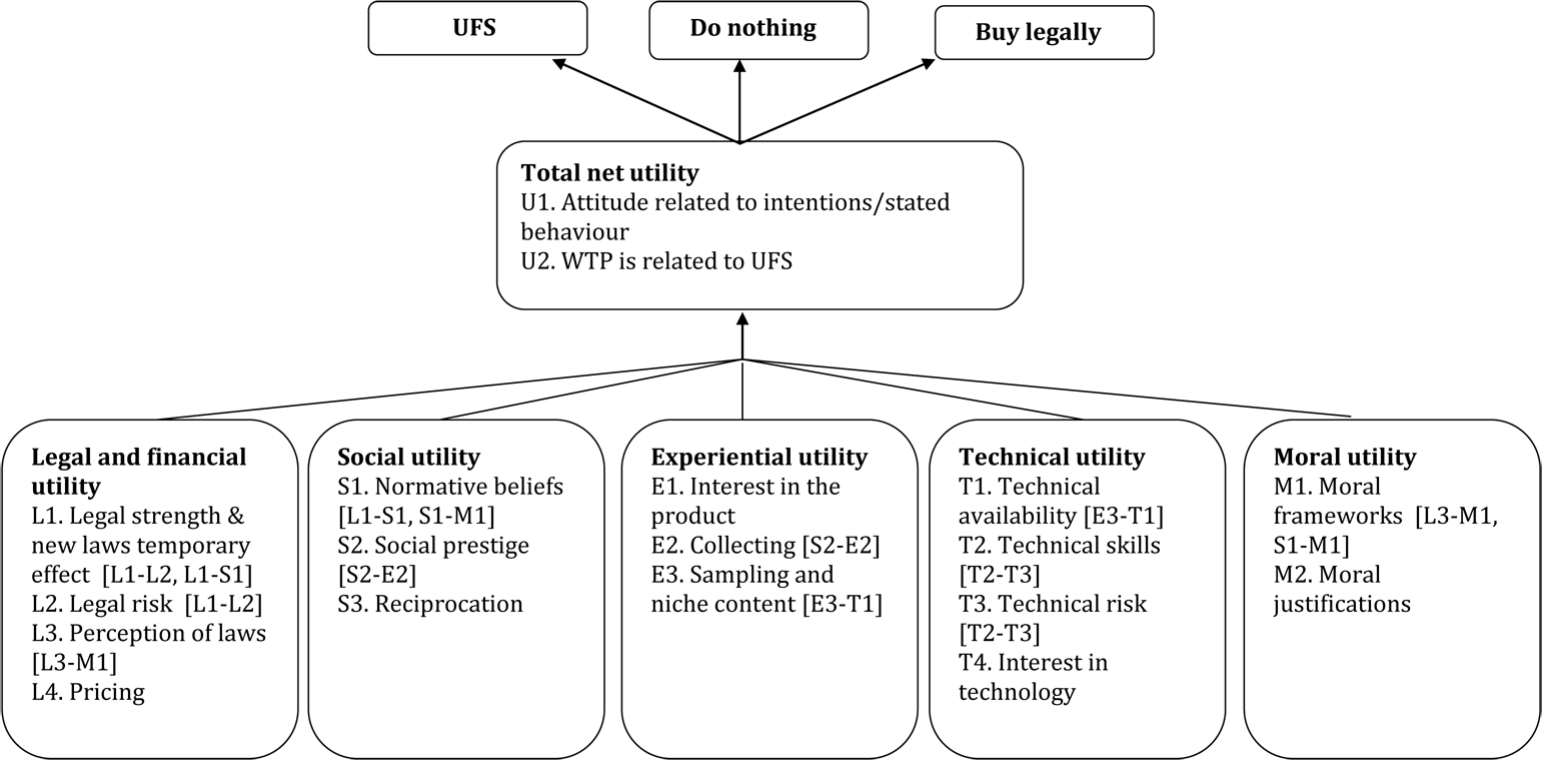
A conceptual framework on the decision to engage in UFS. Note: utility categories are as defined and the links between category elements (in squared brackets) are as described in Table 6.

We have outlined the relevant factors that determine the proposed utilities which the extant literature has identified as predictive of UFS behavior. Fig 3 and Table 6 illustrate the identified factors and their proposed links in a conceptual model.

**Table 6:** Definition of categories and links in conceptual framework on UFS.

Utility		Definition
Total net utility		Overall assessments regarding how beneficial a behavior is (e.g. attitudes or the results of a cost-benefit analysis), or a reported intent to engage in a behavior in future
Legal and financial net utility		Factors associated with financial outlay for legal purchasing as well as the perceived likelihood and legal and financial consequences of detection whilst engaged in unlawful activity, such as monetary fines
Experiential net utility		Factors associated with perceptions of goods themselves such as individuals’ interest in a media type or a desire to experience goods
Technical net utility		Factors associated with individuals’ perceived or actual ability to unlawfully file share, for example their technical skill or the availability of broadband connections
Social net utility		Factors associated with the influence others can have upon the behavior of an individual. For example, whether or not peers engage in unlawful file sharing or perceive the behavior to be acceptable or not
Moral net utility		Factors associated with how right or wrong unlawful file sharing is perceived to be by an individual, and how mismatches between individuals moral beliefs and their actual behaviors behavior are managed
Link	Connects	Description
L1-L2	Legal strength—Legal risk	Stronger legal IP regimens are associated with enhanced legal risks.
L1-S1	Legal strength—Normative beliefs	Different cultures have different beliefs about file sharing, which impact upon legal enforcement.
L3-M1	Perception of laws—Moral frameworks	UFS does not always feel like a crime, and in cultures that consider UFS moral the validity of the laws may questioned rather than the appropriateness of UFS.
S2-E2	Collecting—Social prestige	Having content to provide to others can enhance social prestige as well as increase the library for personal consumption.
E3-T1	Sampling and niche content—Technical availability	The technical availability of niche content via UFS networks allows users to indulge an interest in media not (widely) available legally.
T1-T2	Technical skills—Technical risk	Greater technical skill permits a lower risk of contracting malware and damaging equipment.
S1-M1	Normative beliefs—Moral frameworks	Social norms in part reflect wider cultural moral beliefs.

Note: the links are as defined in Fig 3.

Table 6 defines the different utility categories as emerging from the literature. Under the category of *legal and financial utility*, we have considered the legal strength and risks associated to different legal protection regimes as acting as deterrent of UFS; it appears that stricter laws increase purchasing and decrease UFS, with relevant qualifications discussed in the previous section. We have also considered how the perception of laws and pricing matter. The evidence for actual price effects is only unequivocal regarding purchasing; there is mixed evidence for the effect of price on UFS. At a national level stronger laws decrease UFS but this evidence is confounded by culture and national wealth. There is also evidence that the effect of new laws or lawsuits is only temporary—it may be that people are initially concerned by the risk but quickly habituate or adapt their behavior around the new laws.

Under the category of *social utility*, we have considered the role of moral beliefs, social prestige and reciprocation in creating a social environment conducive to UFS. Social norms can potentially increase UFS. Under the category of *experiential utility*, we have noted how interest in the product, a collecting motive, sampling and niche content may increase UFS. For example, UFS is used to learn about a product. Under the category of *technical utility*, we have noted how the technical availability or otherwise of a legal alternative affects UFS, as do the technical skills and risk; UFS is used to obtain hard to access, unreleased or technically flexible media which are not offered by legal means. UFS increases due to legally unavailable content are maintained even when the content becomes legally available, suggesting some form of habit formation. An interest in technology potentially increases UFS. Under the category of *moral utility*, we have identified the role of moral frameworks and justifications in being consistent or not with UFS. UFS not feeling like a crime is consistent with more UFS. People who base their moral beliefs on the law or other absolutist frameworks view UFS negatively whereas relativist moral frameworks or further considerations such as social welfare have a positive view of UFS.

Crucially, different categories can be expected to interact with one another, i.e. it cannot be presumed that their effect on UFS is linearly additive. Fig 3 identifies a number of links between category elements emerging from our review, and Table 6 lists them and describes them. For example, experiential utility and technical utility may interact in two ways: technical availability facilitates a sampling and niche content seeking motive and, while generally being a disincentive to engage in UFS, technical risks of viruses are less of an issue to more experienced file sharers. As a third example, in this case related to an interaction between moral and legal utility, if UFS does not feel like a crime, this may interact with the perception of laws, as their validity may be questioned rather than the appropriateness of UFS.

The five utility categories combine to provide a total utility value of engaging in UFS, buying legally and doing nothing. We can normalize the total utility from this last option to 0. The consumer will then buy the product legally if he or she gains positive total utility from doing so and this is higher than that from engaging in UFS. Vice versa, he or she will engage in UFS if he or she gains positive total utility from doing so and this is higher than buying legally. The connection between willingness to pay (WTP) and increases in purchasing and decreases in UFS is clear from the existing literature, and there does remain considerable if circumstantial evidence that some decrease in UFS (e.g. due to file sharing website closure) creates some increase for sales. Our model therefore permits the complexity of UFS behavior to be represented in a comparatively simple framework that is grounded in the available empirical data (Ritchie & Spencer, 1994).

Another outcome of this review is the apparent difference across media types. Although some studies treat all media as a single entity, and few studies compare media types, we did observe some media specific effects regarding software. There is evidence that, while music is an inferior good whose sales decrease with income, software is a normal good whose legal sales increase with income. Interest in the product and technical skill was predictive of UFS for most media types except software; software is perceived just be a tool they utilized with no emotional engagement. Internet access decreased physical music sales but increased software sales. This suggests that software acquisition is not elective; that the UFS decision is driven by practical considerations such as affordability and availability. Interestingly no differences were observed between primarily visual or primarily acoustic media, nor between media requiring the relatively small or relatively large data transfers.

### Limitations

The technological landscape is constantly changing and this review is the product of the time in which the research was conducted. Most of the factors studied will remain influential, as people will remain concerned by legal and financial costs and motivated by experiential, moral and social factors. However, technical factors and available media continue to change. The media focus reflects currently popular media. Historically, small music files were most easily bought or file shared and the focus of much of the research. The increasing ability to download or stream large files makes TV and Movie lawful and unlawful streaming services increasingly important. Legal availability is also changing and adapting to consumer demand.

One limitation, observable from Fig 2, is the comparative scarcity of studies that employ observed behavior as a measured outcome, whether from the experimental laboratory or from the natural world. This scarcity is especially severe for moral net utility but also experiential net utility and social net utility. This makes conclusions such as one that stronger IP laws may be ineffective if set against the moral beliefs or social norms of the society (Svennson and Larsson, 2009), virtually impossible to evaluate at present. The reason for the limited use of observed behavior as the measured outcome may reflect on the one side the lack of familiarity with laboratory experimental methods in most of this literature, and on the other side the ethical and technical complexities of observing unlawful behavior in a manner that has little to no risk for participants whilst providing robust empirical evidence. Nonetheless this quality of evidence is necessary if the objective is to state with confidence which variables can be demonstrated to be causally associated with UFS behavior. The focus upon intent to file share as an outcome is also somewhat concerning given that very few studies seek to establish a relationship between intentions and stated behavior, and given that that some variables, including legal deterrents, may impact upon intentions more strongly than they do upon actual behavior.

The second limitation concerns the fact that the vast majority of the studies employ cross sectional surveys, which makes attributions of causality extremely difficult. There is a clear need for longitudinal studies to better determine causality links, e.g. from stated moral beliefs into future behavior while controlling for past behavior.

A third limitation is that the UFS debate seems to have been played out largely, in relation to evidence, in terms of music files. Movies and software are a distant second, and not well represented for all potential net utility sources. There is very little on videogames, books, or TV content, and the pornography market was ruled out from our cubic representation as we found no studies on it. One study does hint at the consequences of ignoring pornography as a media. Mateus and Peha [23] found that, for participants that use p2p networks to access pornography, 96% of users also download other copyrighted material compared to 65% of users that do not use p2p networks to download pornography. Another example of a notable gap is videogames, traditionally seen as a past-time associated with technically savvy younger adults. The extremely limited evidence identified suggests that despite their technical knowhow, videogame players may choose to not file share, or at least do so to a far lesser extent than consumers of other media [90]. This finding requires replication, and, if robust, the reasons for this could be valuable for those seeking to encourage legal sales in other media.

A fourth limitation is the comparatively widespread use of generic estimates of UFS that are not specific to particular media. We have highlighted that the different elements of the proposed utility model may vary in importance depending upon media. Therefore it would appear to be problematic to assume that the motives of UFS are identical between media; given this, the use of generic dependent variables is a limitation to consider.

## Conclusions

We employed a scoping review methodology to consider and assess the existing evidence on the determinants of UFS in as a systematic and transparent way as possible. Of course, research on UFS is continuing beyond the period considered in this scoping review. For example, a recent paper by Poort *et al*. [91] looks at the effects of legally blocking access to the Pirate Bay in the Netherlands and a survey was carried out by Karaganis and Renkema [92] which compared copying in the US and Germany. Neither the paper nor this survey contradict the findings presented in our review.

We presented a conceptual framework that considers the decision of a consumer to engage in unlawful downloading, purchase a legal copy or do nothing, depending on the utilities obtained from five interacting sources: legal and financial, experiential, technical, social and moral utility. This framework enables us to represent the studies on the determinants of UFS along the three dimensions of a ‘cubic’ space, where the dimensions are the net utility source, the market medium and the outcome measure. We find a complex relationship between UFS and purchasing, limitations for legal influence, but alternative social, moral, technical and experiential avenues of influence for which more research would be useful, as would be for media other than music files, and particularly videogames, books, or TV content.

## Supporting Information

S1 File Full list of included studies. (DOCX)

S2 File Data extraction form. (XLSX)
