# Supplementary material for: Determinants of Unlawful File Sharing: A Scoping Review
Source: PLoS One. 2015 Jun 1;10(6):e0127921. doi: 10.1371/journal.pone.0127921 (PMC4452318; doi:10.1371/journal.pone.0127921)
Supplement: S1 File — (DOCX) [file pone.0127921.s001.docx]

# Supplement 1

# Full List of Included Studies

Acılar, A. (2010). Demographic factors affecting freshman students' attitudes towards software piracy: An empirical study. *Issues in Informing Science & Information Technology, 7*, 321-328.

Adermon, A., & Liang, C.-Y. (2011). *Piracy, music, and movies: A natural experiment*. Uppsala Center for Labor Studies. IFN Working Paper No. 854 Retrieved from <http://papers.ssrn.com/sol3/papers.cfm?abstract_id=1752224>

Aguiar, L., & Martens, B. (2013). Digital Music Consumption on the Internet: Evidence from Clickstream Data. from <http://ipts.jrc.ec.europa.eu/publications/pub.cfm?id=6084>

Al-Rafee, S., & Cronan, T. P. (2006). Digital piracy: Factors that influence attitude toward behavior. *Journal of Business Ethics, 63*(3), 237-259. doi: 10.1007/s10551-005-1902-9

Al-Rafee, S., & Dashti, A. E. (2012). A cross cultural comparison of the extended TPB: The case of digital piracy. *Journal of Global Information Technology Management, 15*(1), 5-24. doi: 10.1080/1097198X.2012.10845610

Al-Rafee, S., & Rouibah, K. (2010). The fight against digital piracy: An experiment. *Telematics & Informatics, 27*(3), 283-292. doi: 10.1016/j.tele.2009.12.002

Aleassa, H., Pearson, J. M., & McClurg, S. (2011). Investigating software piracy in jordan: An extension of the theory of reasoned action. *Journal of Business Ethics, 98*(4), 663-676. doi: 10.1007/s10551-010-0645-4

Allen, P. J., Shepherd, K. L., & Roberts, L. D. (2010). Peer-to-peer file-sharing: Psychological reactance and the Theory of Planned Behaviour. *International Journal of Technoethics, 1*(4), 49-64. doi: 10.4018/jte.2010100104

Altschuller, S., & Benbunan-Fich, R. (2009). Is music downloading the new prohibition? What students reveal through an ethical dilemma. *Ethics and Information Technology, 11*(1), 49-56. doi: 10.1007/s10676-008-9179-1

Andersen, B., & Frenz, M. (2010). Don't blame the P2P file-sharers: the impact of free music downloads on the purchase of music CDs in Canada. *Journal of Evolutionary Economics, 20*(5), 715-740. doi: 10.1007/s00191-010-0173-5

Bahanovich, D., & Collopy, D. (2009). Music experience and behaviour in young people: University of Hertfordshire, UK.

Bai, J., & Waldfogel, J. (2012). Movie piracy and sales displacement in two samples of chinese consumers. *Information Economics and Policy, 24*(3-4), 187-196. doi: <http://www.sciencedirect.com/science/journal/01676245>

Balducci, F. (2009). Music or hi-tech lovers? Inferring into the determinants of music consumption. *Rivista Italiana degli Economisti, 14*(2), 361-390. doi: 10.1427/30395

Beekhuyzen, J., von Hellens, L., & Nielsen, S. (2011). Underground online music communities: Exploring rules for membership. *Online Information Review, 35*(5), 699-715. doi: 10.1108/14684521111176453

Bellemare, M., & Holmberg, A. (2010). *The determinants of music piracy in a sample of college students*. University of Minnesota. Retrieved from <http://papers.ssrn.com/sol3/papers.cfm?abstract_id=1481272>

Bhal, K. T., & Leekha, N. D. (2008). Exploring cognitive moral logics using grounded theory: The case of software piracy. *Journal of Business Ethics, 81*(3), 635-646. doi: 10.1007/s10551-007-9537-7

Bhattacharjee, S., Gopal, R. D., Lertwachara, K., & Marsden, J. R. (2006). Impact of legal threats on online music sharing activity: An analysis of music industry legal actions. *Journal of Law & Economics, 49*(1), 91-114. doi: 10.1086/501085

Bhattacharjee, S., Gopal, R. D., Lertwachara, K., Marsden, J. R., & Telang, R. (2007). The effect of digital sharing technologies on music markets: A survival analysis of albums on ranking charts. *Management Science, 53*(9), 1359-1374. doi: 10.1287/mnsc.1070.0699

Blackburn, D. (2005). *Essays on the economics of copying with an application to the recorded music industry.* (Doctoral Dissertation), Harvard University. Retrieved from <http://search.ebscohost.com/login.aspx?direct=true&db=eoh&AN=0874089&site=ehost-live> Available from EBSCOhost eoh database.

BMRB Social Research. (2009). Future copyright development: Intellectual Property Office, UK.

Bonner, S., & O'Higgins, E. (2010). Music piracy: ethical perspectives. *Management Decision, 48*(9), 1341-1354. doi: 10.1108/00251741011082099

Boorstin, E. S. (2004). *Music sales in the age of file sharing.* (PhD), Princeton University. Retrieved from <http://www.cs.princeton.edu/~felten/boorstin-thesis.pdf>

Bounie, D., Bourreau, M., & Waelbroeck, P. (2006). Piracy and the demand for films: Analysis of piracy behavior in french universities. *Review of Economic Research on Copyright Issues, 3*(2), 15-27. doi: <http://www.serci.org/default.asp>

Bounie, D., Bourreau, M., & Waelbroeck, P. (2007). Pirates or explorers? Analysis of music consumption in french graduate schools. *Brussels Economic Review/Cahiers Economiques de Bruxelles, 50*(2), 167-192. doi: <http://homepages.vub.ac.be/~mcincera/BER/BER.html>

Buxmann, P., Pohl, G., Johnscher, P., & Strube, J. (2005). Strategies for digital music markets: Pricing and the effectiveness of measures against pirate copies. *Wirtschaftsinformatik, 47*(2), 118-125.

Cenite, M., Wang, M. W., Peiwen, C., & Chan, G. S. (2009). More than just free content: Motivations of peer-to-peer file sharers. *Journal of Communication Inquiry, 33*(3), 206-221. doi: 10.1177/0196859909333697

Chan, R. Y. K., & Lai, J. W. M. (2011). Does ethical ideology affect software piracy attitude and behaviour? An empirical investigation of computer users in China. *European Journal of Information Systems, 20*(6), 659-673. doi: 10.1057/ejis.2011.31

Chen, M.-F., Pan, C.-T., & Pan, M.-C. (2009). The joint moderating impact of moral intensity and moral judgment on consumer's use intention of pirated software. *Journal of Business Ethics, 90*(3), 361-373. doi: 10.1007/s10551-009-0046-8

Chen, M.-F., & Yen, Y.-H. (2011). Costs and utilities perspective of consumers' intentions to engage in online music sharing: Consumers' knowledge matters. *Ethics & Behavior, 21*(4), 283-300. doi: 10.1080/10508422.2011.585595

Chen, Y., & Jianming, Z. (2007). *The effect of internet on consumer's pirated behavior in China*. Paper presented at the Sixth Wuhan International Conference on E-Business, Vols 1-4: Management Challenges in a Global World, Wuhan, China. <Go to ISI>://WOS:000249025702038

Chen, Y.-C., Shang, R.-A., & Lin, A.-K. (2008). The intention to download music files in a P2P environment: Consumption value, fashion, and ethical decision perspectives. *Electronic Commerce Research and Applications, 7*(4), 411-422. doi: 10.1016/j.elerap.2008.02.001

Chiang, E. P., & Assane, D. (2007). Determinants of music copyright violations on the university campus. *Journal of Cultural Economics, 31*(3), 187-204. doi: 10.1007/s10824-007-9042-y

Chiang, E. P., & Assane, D. (2008). Music piracy among students on the university campus: Do males and females react differently? *The Journal of Socio-Economics, 37*(4), 1371-1380. doi: 10.1016/j.socec.2007.08.011

Chiang, E. P., & Assane, D. (2009). Estimating the willingness to pay for digital music. *Contemporary Economic Policy, 27*(4), 512-522. doi: <http://www.blackwell-synergy.com/loi/coep/>

Chiou, J.-S., Cheng, H.-I., & Huang, C.-Y. (2011). The effects of artist adoration and perceived risk of getting caught on attitude and intention to pirate music in the United States and Taiwan. *Ethics & Behavior, 21*(3), 182-196. doi: 10.1080/10508422.2011.570163

Chiou, J. S., Huang, C. Y., & Lee, H. H. (2005). The antecedents of music piracy attitudes and intentions. *Journal of Business Ethics, 57*(2), 161-174. doi: 10.1007/s10551-004-5263-6

Chiu, H.-C., Hsieh, Y.-C., & Wang, M.-C. (2008). How to encourage customers to use legal software. *Journal of Business Ethics, 80*(3), 583-595. doi: 10.1007/s10551-007-9456-7

Chullasang, N., & Wongpinunwatana, N. (2009). *Individual factors impact on pirating digital media in Thailand*. Paper presented at the Proceedings of the 8th European Conference on Information Warfare and Security, Braga, Portugal. <Go to ISI>://WOS:000280755700005

Cockrill, A., & Goode, M. M. (2012). DVD pirating intentions: Angels, devils, chancers and receivers. *Journal of Consumer Behaviour, 11*(1), 1-10. doi: 10.1002/cb.357

Cox, J., Collins, A., & Drinkwater, S. (2010). Seeders, leechers and social norms: Evidence from the market for illicit digital downloading. *Information Economics and Policy, 22*(4), 299-305. doi: 10.1016/j.infoecopol.2010.09.004

Coyle, J. R., Gould, S. J., Gupta, P., & Gupta, R. (2009). "To buy or to pirate": The matrix of music consumers' acquisition-mode decision-making. *Journal of Business Research, 62*(10), 1031-1037. doi: 10.1016/j.jbusres.2008.05.002

Cronan, T. P., & Al-Rafee, S. (2008). Factors that influence the intention to pirate software and media. *Journal of Business Ethics, 78*(4), 527-545. doi: 10.1007/s10551-007-9366-8

d'Astous, A., Colbert, F., & Montpetit, D. (2005). Music piracy on the web--how effective are anti-piracy arguments? Evidence from the Theory of Planned Behaviour. *Journal of Consumer Policy, 28*(3), 289-310. doi: <http://www.springerlink.com/link.asp?id=400283>

Danaher, B., Dhanasobhon, S., Smith, M. D., & Telang, R. (2010). Converting pirates without cannibalizing purchasers: The impact of digital distribution on physical sales and internet piracy. *Marketing Science, 29*(6), 1138-1151. doi: 10.1287/mksc.1100.0600

Danaher, B., & Smith, M. (2013). *Gone in 60 seconds: The impact of the megaupload shutdown on movie sales*. Wellesley College. March 2013. Retrieved from <http://papers.ssrn.com/sol3/papers.cfm?abstract_id=2229349>

Danaher, B., Smith, M., Telang, R., & Chen, S. (2012). *The effect of graduated response anti-piracy laws on music sales: evidence from an event study in France*. Wellesley College. January 21st 2012. Retrieved from <http://papers.ssrn.com/sol3/papers.cfm?abstract_id=1989240>

Danaher, B., & Waldfogel, J. (2012). *Reel piracy: The effect of online film piracy on international box office sales*. Wellesley College. January 16th, 2012. Retrieved from <http://papers.ssrn.com/sol3/papers.cfm?abstract_id=1986299>

Denegri-Knott, J. (2004). Sinking the online "music pirates": Foucault, power and deviance on the web. *Journal of Computer-Mediated Communication, 9*(4), No Pagination Specified. doi: 10.1111/j.1083-6101.2004.tb00293.x

Depoorter, B., Van Hiel, A., & Vanneste, S. (2011). Copyright backlash. *Southern California Law Review, 84*(6), 1251-1292.

Djekic, P., & Loebbecke, C. (2005). *Software piracy prevention through digital rights management systems*. Paper presented at the CEC 2005: Seventh IEEE International Conference on E-Commerce Technology, Proceedings, Munich, Germany. <http://ieeexplore.ieee.org/xpl/articleDetails.jsp?arnumber=1524097>

Fetscherin, M. (2009). Importance of cultural and risk aspects in music piacy: A cross-national comparison among university students. *Journal of Electronic Commerce Research, 10*(1), 42-55.

Fetscherin, M., Kaskiris, C., & Wallenberg, F. (2005). *Gaming or sharing at LAN-Parties - What is going on?* Paper presented at the First International Conference on Automated Production of Cross Media Content for Multi-channel Distribution, Proceedings, Florence, Italy.

Fetscherin, M., & Lattemann, C. (2007). *Motives and willingness to pay for digital music*. Paper presented at the Axmedis 2007: Third International Conference on Automated Production of Cross Media Content for Multi-Channel Distribution, Barcelona, Spain. <http://ieeexplore.ieee.org/stamp/stamp.jsp?tp=&arnumber=4402876>

Filiciak, M., Hofmokl, J., & Tarkowski, A. (2012). *The circulations of culture - on social distribution of content*. Warsaw School of Social Sciences and Humanities. September 1st, 2012. Retrieved from <http://papers.ssrn.com/sol3/papers.cfm?abstract_id=2246508>

Forman, A. E. (2009). *An exploratory study on the factors associated with ethical intention of digital piracy.* (PhD), Nova Southeastern University. Retrieved from <http://dl.acm.org/citation.cfm?id=1835180> Available from Ovid Technologies PsycINFO database. (12-A)

Frank N. Magid Associates. (2009). Introducing Hollywood’s best customers: Vuze user vs. general internet: Comparative data. from <http://www.magid.com/sites/default/files/pdf/vuze.pdf>

Fukugawa, N. (2011). How serious is piracy in the videogame industry? *Empirical Economics Letters, 10*(3), 225-233. doi: <http://www.eel.my100megs.com/>

Gan, L. L., & Koh, H. C. (2006). An empirical study of software piracy among tertiary institutions in Singapore. *Information & Management, 43*(5), 640-649. doi: 10.1016/j.im.2006.03.005

Garbharran, A., & Thatcher, A. (2012). The impact of occupational field of expertise on intention to pirate software. *International Journal of Psychology, 47*(s1), 404-404. doi: 10.1080/00207594.2012.709107

García-Álvarez, E., López-Sintas, J., & Zerva, K. (2009). A contextual theory of accessing music: Consumer behavior and ethical arguments. *Consumption, Markets & Culture, 12*(3), 243-264. doi: 10.1080/10253860903063253

Garcia-Bardidia, R., Nau, J.-P., & Remy, E. (2011). Consumer resistance and anti-consumption: Insights from the deviant careers of French illegal downloaders. *European Journal of Marketing, 45*(11-12), 1789-1798.

Goles, T., Jayatilaka, B., George, B., Parsons, L., Chambers, V., Taylor, D., & Brune, R. (2008). Softlifting: Exploring determinants of attitude. *Journal of Business Ethics, 77*(4), 481-499. doi: 10.1007/s10551-007-9361-0

Grolleau, G., Mzoughi, N., & Sutan, A. (2008). Please do not pirate it, you will rob the poor! An experimental investigation on the effect of charitable donations on piracy. *The Journal of Socio-Economics, 37*(6), 2417-2426. doi: 10.1016/j.socec.2008.02.001

Gunter, W. D. (2009). *Piracy of the new millennium: An application of criminological theories to digital piracy.* (PhD), University of Delaware. Retrieved from <http://ovidsp.ovid.com/ovidweb.cgi?T=JS&CSC=Y&NEWS=N&PAGE=fulltext&D=psyc6&AN=2010-99050-181>Available from Ovid Technologies PsycINFO database.

Gupta, P. B., Gould, S. J., & Pola, B. (2004). "To pirate or not to pirate": A comparative study of the ethical versus other influences on the consumer's software acquisition-mode decision. *Journal of Business Ethics, 55*(3), 255-274. doi: 10.1007/s10551-004-0991-1

Hadopi. (2011, 23rd january 2011). Hadopi, cultural assets and internet use: practices and perceptions of French internet users. from <http://www.hadopi.fr/download/HADOPI_T0_version_long.pdf>

Haigh, M. (2009). Of ducks and downloads the moral economy of intellectual property in post-soviet society. *Libri, 59*(4), 248-258. doi: 10.1515/libr.2009.022

Hansen, J. M., & Walden, E. A. (2012). *The role of restrictiveness of use in determining ethical and legal awareness of unauthorized file sharing*. Journal of the Association for Information Systems. University of North Carolina at Charlotte. SSRN. Retrieved from <http://ssrn.com/abstract=1311988> or <http://dx.doi.org/10.2139/ssrn.1311988>

Hashim, M., Kannan, K., Maximiano, S., & Rees, J. (2012). *Digital piracy, teens, and the source of advice: An experimental study*. University of Arizona. February 10, 2012. Retrieved from <http://papers.ssrn.com/sol3/papers.cfm?abstract_id=2003106>

Hennig-Thurau, T., Henning, V., & Sattler, H. (2007). Consumer file sharing of motion pictures. *Journal of Marketing, 71*(4), 1-18. doi: 10.1509/jmkg.71.4.1

Hietanen, H., & Räsänen, P. (2009). *Reasons Affecting Frequency of File-Sharing among Finnish Internet Users*. Helsinki Institute for Information Technology. June, 2009. Retrieved from <http://papers.ssrn.com/sol3/papers.cfm?abstract_id=1414209>

Higgins, G. E. (2005). Can low self-control help with the understanding of the software piracy problem? *Deviant Behavior, 26*(1), 1-24. doi: 10.1080/01639620490497947

Higgins, G. E. (2007). Digital piracy: An examination of low self-control and motivation using short-term longitudinal data. *Cyberpsychology & Behavior, 10*(4), 523-529. doi: 10.1089/cpb.2007.9995

Higgins, G. E., Fell, B. D., & Wilson, A. L. (2007). Low self-control and social learning in understanding students' intentions to pirate movies in the United States. *Social Science Computer Review, 25*(3), 339-357. doi: 10.1177/0894439307299934

Higgins, G. E., & Makin, D. A. (2004). Self-control, deviant peers, and software piracy. *Psychological Reports, 95*(3), 921-931. doi: 10.2466/pr0.95.3.921-931

Higgins, G. E., Marcum, C. D., Freiburger, T. L., & Ricketts, M. L. (2012). Examining the role of peer influence and self-control on downloading behavior. *Deviant Behavior, 33*(5), 412-423. doi: 10.1080/01639625.2011.584275

Higgins, G. E., Wolfe, S. E., & Marcum, C. D. (2008). Digital piracy: An examination of three measurements of self-control. *Deviant Behavior, 29*(5), 440-460. doi: 10.1080/01639620701598023

Higgins, G. E., Wolfe, S. E., & Ricketts, M. L. (2009). Digital piracy a latent class analysis. *Social Science Computer Review, 27*(1), 24-40. doi: 10.1177/0894439308321350

Hinduja, S., & Higgins, G. E. (2011). Trends and patterns among music pirates. *Deviant Behavior, 32*(7), 563-588. doi: 10.1080/01639625.2010.514202

Hinduja, S., & Ingram, J. R. (2009). Social learning theory and music piracy: The differential role of online and offline peer influences. *Criminal Justice Studies: A Critical Journal of Crime, Law & Society, 22*(4), 405-420. doi: 10.1080/14786010903358125

Holt, T. J., Bossler, A. M., & May, D. C. (2012). Low self-control, deviant peer associations, and juvenile cyberdeviance. *American Journal of Criminal Justice, 37*(3), 378-395. doi: 10.1007/s12103-011-9117-3

Holt, T. J., & Copes, H. (2010). Transferring subcultural knowledge on-line: Practices and beliefs of persistent digital pirates. *Deviant Behavior, 31*(7), 625-654. doi: 10.1080/01639620903231548

Hsu, J. L., & Shiue, C. W. (2008). Consumers' willingness to pay for non-pirated software. *Journal of Business Ethics, 81*(4), 715-732. doi: 10.1007/s10551-007-9543-9

Hsu, J. L., & Su, Y.-L. (2008). Usage of unauthorized software in Taiwan. *Social Behavior and Personality, 36*(1), 1-8. doi: 10.2224/sbp.2008.36.1.1

Huang, C. Y. (2005). File sharing as a form of music consumption. *International Journal of Electronic Commerce, 9*(4), 37-55.

Huang, M., Zhu, H., & Liu, M. (2007). *Why do chinese users use pirated software: An integrative perspective?* Paper presented at the 2007 International Conference on Wireless Communications, Networking and Mobile Computing, Vols 1-15, Shanghai. China. <http://ieeexplore.ieee.org/stamp/stamp.jsp?arnumber=4341218>

Huygen, A., Helberger, N., Poort, J., Rutten, P., & Van Eijk, N. (2009). Ups and downs; economic and cultural effects of file sharing on music, film and games *TNO Information and Communication Technology Series*: IViR.

Ingram, J. R., & Hinduja, S. (2008). Neutralizing music piracy: an empirical examination. *Deviant Behavior, 29*(4), 334-366. doi: 10.1080/01639620701588131

Jacobs, R. S., Heuvelman, A., Tan, M., & Peters, O. (2012). Digital movie piracy: A perspective on downloading behavior through social cognitive theory. *Computers in Human Behavior, 28*(3), 958-967. doi: 10.1016/j.chb.2011.12.017

Jambon, M. M., & Smetana, J. G. (2012). College students' moral evaluations of illegal music downloading. *Journal of Applied Developmental Psychology, 33*(1), 31-39. doi: 10.1016/j.appdev.2011.09.001

Karakaya, M. (2011). *Analysis of the key reasons behind the pirated software usage of Turkish Internet users: Application of routine activities theory.* (Doctor of Communications Design), University of Baltimore. Available from Ovid Technologies PsycINFO database.

Ki, E.-J., Chang, B.-H., & Khang, H. (2006). Exploring influential factors on music piracy across countries. *Journal of Communication, 56*(2), 406-426. doi: 10.1111/j.1460-2466.2006.00026.x

Kini, R. B., Ramakrishna, H., & Vijayaraman, B. (2003). An exploratory study of moral intensity regarding software piracy of students in Thailand. *Behaviour & Information Technology, 22*(1), 63-70. doi: 10.1080/01449290301784

Kinnally, W., Lacayo, A., McClung, S., & Sapolsky, B. (2008). Getting up on the download: College students' motivations for acquiring music via the web. *New Media & Society, 10*(6), 893-913. doi: 10.1177/1461444808096250

Konstantakis, N. I., Palaigeorgiou, G. E., Siozos, P. D., & Tsoukalas, I. A. (2010). What do computer science students think about software piracy? *Behaviour & Information Technology, 29*(3), 277-285. doi: 10.1080/01449290902765076

Kwan, S. S. K. (2008). *End-user digital piracy: Contingency framework, affective determinants and response distortion.* (PhD), Hong Kong University of Science and Technology. Available from Ovid Technologies PsycINFO database.

Kwong, S. W., & Park, J. (2008). Digital music services: consumer intention and adoption. *Service Industries Journal, 28*(10), 1463-1481. doi: 10.1080/02642060802250278

Lai, M., & Kuo, C.-C. (2007). Preventing piracy use intention by rectifying self-positivity bias. *Social Behavior and Personality, 35*(7), 961-974. doi: 10.2224/sbp.2007.35.7.961

LaRose, R., & Kim, J. (2007). Share, steal, or buy? A social cognitive perspective of music downloading. *Cyberpsychology & Behavior, 10*(2), 267-277. doi: 10.1089/cpb.2006.9959

LaRose, R., Lai, Y. J., Lange, R., Love, B., & Wu, Y. (2005). Sharing or piracy? An exploration of downloading behavior. *Journal of Computer-Mediated Communication, 11*(1), 1-21. doi: 10.1111/j.1083-6101.2006.00001.x

Lee, D., Park, J. Y., Kim, J., Kim, J., & Moon, J. (2011). Understanding music sharing behaviour on social network services. *Online Information Review, 35*(5), 716-733. doi: 10.1108/14684521111176462

Lee, S. (2006). *The effect of file sharing on consumer’s purchasing pattern: A survey approach.* Paper presented at the Telecommunications Policy Research Conference, Washington DC, USA.

Leung, T. C. (2012). *Music piracy: Bad for record sales but good for the iPod?* The Chinese University of Hong Kong. October 2012. Retrieved from <http://papers.ssrn.com/sol3/papers.cfm?abstract_id=2244111>

Levin, A. M., Dato-on, M. C., & Manolis, C. (2007). Deterring illegal downloading: The effects of threats appeals, past behavior, subjective norms, and attributions of harm. *Journal of Consumer Behaviour, 6*(2-3), 111-122. doi: 10.1002/cb.211

Liao, C., Lin, H.-N., & Liu, Y.-P. (2010). Predicting the use of pirated software: A contingency model integrating perceived risk with the Theory of Planned Behavior. *Journal of Business Ethics, 91*(2), 237-252. doi: 10.1007/s10551-009-0081-5

Liebowitz, S. J. (2008). Testing file sharing's impact on music album sales in cities. *Management Science, 54*(4), 852-859. doi: 10.1287/mnsc.1070.0833

Limayem, M., Khalifa, M., & Chin, W. W. (2004). Factors motivating software piracy: A longitudinal study. *Ieee Transactions on Engineering Management, 51*(4), 414-425. doi: 10.1109/tem.2004.835087

Long, X. (2011). Intellectual property rights protection and recorded music sales: Focus on 26 OECD countries panel data. *Frontiers of Economics in China, 6*(2), 211-228. doi: 10.1007/s11459-011-0129-1

Lu, J. (2009). Chinese culture and software copyright. *New Media & Society, 11*(8), 1372-1393. doi: 10.1177/1461444809341262

Lysonski, S., & Durvasula, S. (2008). Digital piracy of MP3s: Consumer and ethical predispositions. *Journal of Consumer Marketing, 25*(3), 167-178. doi: 10.1108/07363760810870662

Ma, L., Montgomery, A., Singh, P., & Smith, M. (2011). *The Effect of Pre-Release Movie Piracy on Box-Office Revenue*. Carnegie Mellon University. March 2011. Retrieved from <http://papers.ssrn.com/sol3/papers.cfm?abstract_id=1782924>

Maffioletti, A., & Ramello, G. B. (2004). Should we put them in jail? Copyright infringement, penalties and consumer behaviour: Insights from experimental data. *Review of Economic Research on Copyright Issues, 1*(2), 81-95. doi: <http://www.serci.org/default.asp>

Malin, J., & Fowers, B. J. (2009). Adolescent self-control and music and movie piracy. *Computers in Human Behavior, 25*(3), 718-722. doi: 10.1016/j.chb.2008.12.029

Mandel, P., & Suessmuth, B. (2012). Determinants of digital piracy: A re-examination of results. *Jahrbucher Fur Nationalokonomie Und Statistik, 232*(4), 394-413.

Mateus, A. M., & Peha, J. M. (2008). *Dimensions of P2P and digital piracy in a university campus*. Paper presented at the Proceedings of 2008 Telecommunications Policy Research Conference, Arlington, VA (USA). <http://repository.cmu.edu/epp/23/>

Michel, N. J. (2005). Digital file sharing and the music industry: Was there a substitution effect? *Review of Economic Research on Copyright Issues, 2*(2), 41-52. doi: <http://www.serci.org/default.asp>

Montoro Pons, J. d. D., & Cuadrado Garcia, M. (2008). Legal origin and intellectual property rights: an empirical study in the prerecorded music sector. *European Journal of Law and Economics, 26*(2), 153-173. doi: 10.1007/s10657-008-9056-8

Montoro-Pons, J. D., & Cuadrado-García, M. (2006). *Digital goods and the effects of copying: an empirical study of the music market.* Paper presented at the 14th International Conference on Cultural Economics, Vienna, Austria.

Moores, T. T. (2010). Untangling the web of relationships between wealth, culture, and global software piracy rates: A path model. *Journal of Global Information Management, 18*(1), 1-14. doi: 10.4018/jgim.2010091103

Moores, T. T., Nill, A., & Rothenberger, M. A. (2009). Knowledge of software piracy as an antecedent to reducing pirating behavior. *Journal of Computer Information Systems, 50*(1), 82-89.

Morris, R. G., & Higgins, G. E. (2009). Neutralizing potential and self-reported digital piracy: A multitheoretical exploration among college undergraduates. *Criminal Justice Review, 34*(2), 173-195. doi: 10.1177/0734016808325034

Morris, R. G., Johnson, M. C., & Higgins, G. E. (2009). The role of gender in predicting the willingness to engage in digital piracy among college students. *Criminal Justice Studies: A Critical Journal of Crime, Law & Society, 22*(4), 393-404. doi: 10.1080/14786010903358117

Morton, N. A., & Koufteros, X. (2008). Intention to commit online music piracy and its an empirical antecedents: An empirical investigation. *Structural Equation Modeling-a Multidisciplinary Journal, 15*(3), 491-512. doi: 10.1080/10705510802154331

Mun, S.-H. (2008). *Culture-related aspects of intellectual property rights: A cross-cultural analysis of copyright.* (PhD), University of Texas at Austin. Retrieved from <http://repositories.lib.utexas.edu/handle/2152/17888?show=full> Available from Ovid Technologies PsycINFO database.

Nandi, T. K., & Rochelandet, F. (2008). The incentives for contributing digital contents over p2p networks: An empirical investigation. *Review of Economic Research on Copyright Issues, 5*(2), 19-35. doi: <http://www.serci.org/default.asp>

North, A. C., & Oishi, A. (2006). Music CD purchase decisions. *Journal of Applied Social Psychology, 36*(12), 3043-3084. doi: 10.1111/j.0021-9029.2006.00142.x

Nunes, J. C., Hsee, C. K., & Weber, E. U. (2004). Why are people so prone to steal software? The effect of cost structure on consumer purchase and payment intentions. *Journal of Public Policy & Marketing, 23*(1), 43-53. doi: 10.1509/jppm.23.1.43.30398

Oestreicher-Singer, G., & Sundararajan, A. (2010). *Are digital rights valuable? Theory and evidence from ebook pricing*. Tel Aviv University. June 2010. Retrieved from <http://papers.ssrn.com/sol3/papers.cfm?abstract_id=871243>

Ofcom. (2011). Qualitative research into online digital piracy. from <http://stakeholders.ofcom.org.uk/binaries/research/telecoms-research/filesharing/gfk.pdf>

Ofcom. (2012). OCI Tracker Benchmark Study Q3 2012. from <http://stakeholders.ofcom.org.uk/binaries/research/telecoms-research/online-copyright/Kantar-Media.pdf>

Papies, D., & Clement, M. (2008). Adoption of new movie distribution services on the internet. *Journal of Media Economics, 21*(3), 131-157. doi: 10.1080/08997760802300530

Peace, A. G., Galletta, D. F., & Thong, J. Y. L. (2003). Software piracy in the workplace: A model and empirical test. *Journal of Management Information Systems, 20*(1), 153-177.

Pearce, K. E. (2011). Convergence through mobile peer-to-peer file sharing in the republic of armenia. *International Journal of Communication, 5*, 511-528.

Peitz, M., & Waelbroeck, P. (2004). *The effect of internet piracy on CD sales: Cross-section evidence*. University of Mannheim. January 2004. Retrieved from <http://papers.ssrn.com/sol3/papers.cfm?abstract_id=511763>

Pénard, T., Dejean, S., & Suire, R. (2010). *Olson’s Paradox Revisited: An Empirical Analysis of Incentives to Contribute in P2P File-sharing Communities*. Center for Research in Economics and Management (CREM), University of Rennes 1, University of Caen and CNRS. July 1, 2010. Retrieved from <http://papers.ssrn.com/sol3/papers.cfm?abstract_id=1299190>

Pertierra, A. C. (2012). If they show prison break in the united states on a Wednesday, by Thursday it is here: Mobile media networks in twenty-first-century Cuba. *Television & New Media, 13*(5), 399-414. doi: 10.1177/1527476412443564

Phau, I., & Liang, J. (2012). Downloading digital video games: Predictors, moderators and consequences. *Marketing Intelligence & Planning, 30*(7), 740-756. doi: 10.1108/02634501211273832

Phau, I., & Ng, J. (2010). Predictors of usage intentions of pirated software. *Journal of Business Ethics, 94*(1), 23-37. doi: 10.1007/s10551-009-0247-1

Plouffe, C. R. (2008). Examining "peer-to-peer" (P2P) systems as consumer-to-consumer (C2C) exchange. *European Journal of Marketing, 42*(11-12), 1179-1202. doi: 10.1108/03090560810903637

Plowman, S., & Goode, S. (2009). Factors affecting the intention to download music: Quality perceptions and downloading intensity. *Journal of Computer Information Systems, 49*(4), 84-97.

Poort, J., & Leenheer, J. (2012). File sharing 2©12: Downloading from illegal sources in the Netherlands: IViR.

Robertson, K., McNeill, L., Green, J., & Roberts, C. (2012). Illegal downloading, ethical concern, and illegal behavior. *Journal of Business Ethics, 108*(2), 215-227. doi: 10.1007/s10551-011-1079-3

Rochelandet, F., & Le Guel, F. (2005). P2P music sharing networks: Why the legal fight against copiers may be inefficient. *Review of Economic Research on Copyright Issues, 2*(2), 69-82. doi: <http://www.serci.org/default.asp>

Sandulli, F. D. (2007). CD music purchase behaviour of P2P users. *Technovation, 27*(6-7), 325-334. doi: 10.1016/j.technovation.2006.12.007

Sandulli, F. D., & Martin-Barbero, S. (2007). 68 Cents per song. *Convergence: The Journal of Research into New Media Technologies, 13*(1), 63-78. doi: 10.1177/1354856507072857

Sandulli, F. D., & Martín-Barbero, S. (2006). 99 cents per song: A fair price for digital music? The effects of music industry strategies to raise the willingness to pay by P2P users. *Journal of Website Promotion, 2*(3/4), 3-15. doi: 10.1080/15533610802174888

Shanahan, K. J., & Hyman, M. R. (2010). Motivators and enablers of SCOURing: A study of online piracy in the US and UK. *Journal of Business Research, 63*(9-10), 1095-1102. doi: 10.1016/j.jbusres.2009.02.026

Shang, R.-A., Chen, Y.-C., & Chen, P.-C. (2008). Ethical decisions about sharing music files in the P2P environment. *Journal of Business Ethics, 80*(2), 349-365. doi: 10.1007/s10551-007-9424-2

Sheehan, B., Tsao, J., & Pokrywczynski, J. (2012). Stop the music! How advertising can help stop college students from downloading music illegally. *Journal of Advertising Research, 52*(3), 309-321. doi: 10.2501/jar-52-3-309-321

Sheehan, B., Tsao, J., & Yang, S. (2010). Motivations for gratifications of digital music piracy among college students. *Atlantic Journal of Communication, 18*(5), 241-258. doi: 10.1080/15456870.2010.521471

Sinha, R. K., Machado, F. S., & Sellman, C. (2010). Don't think twice, it's all right: Music piracy and pricing in a DRM-free environment. *Journal of Marketing, 74*(2), 40-54. doi: 10.1509/jmkg.74.2.40

Sinha, R. K., & Mandel, N. (2008). Preventing digital music piracy: The carrot or the stick? *Journal of Marketing, 72*(1), 1-15. doi: 10.1509/jmkg.72.1.1

Siponen, M., Vance, A., & Willison, R. (2012). New insights into the problem of software piracy: The effects of neutralization, shame, and moral beliefs. *Information & Management, 49*(7-8), 334-341. doi: 10.1016/j.im.2012.06.004

Siponen, M., & Vartiainen, T. (2005). Attitudes to and factors affecting unauthorized copying of computer software in Finland. *Behaviour & Information Technology, 24*(4), 249-257. doi: 10.1080/01449290512331321857

Smith, M. D., & Telang, R. (2010). Piracy or promotion? The impact of broadband Internet penetration on DVD sales. *Information Economics and Policy, 22*(4), 289-298. doi: 10.1016/j.infoecopol.2010.02.001

Steinmetz, K. F., & Tunnell, K. D. (2013). Under the pixelated jolly roger: A study of on-line pirates. *Deviant Behavior, 34*(1), 53-67. doi: 10.1080/01639625.2012.707536

Sung, T.-W. (2007). *An economic analysis of new peer-to-peer transfer activities.* (PhD), Claremont Graduate University. Retrieved from <http://search.ebscohost.com/login.aspx?direct=true&db=eoh&AN=1002750&site=ehost-live> Available from EBSCOhost eoh database.

Svensson, M., & Larsson, S. (2009). *Social norms and intellectual property. Online norms and the European legal development*. Research Report in Sociology of Law, Vol. 1. Lund University. Retrieved from https://lup.lub.lu.se/search/publication/1510388

Svensson, M., & Larsson, S. (2012). Intellectual property law compliance in Europe: Illegal file sharing and the role of social norms. *New Media & Society, 14*(7), 1147-1163. doi: 10.1177/1461444812439553

Tanaka, T. (2004). *Does file sharing reduce music CD sales?: A case of Japan version 0.1*. Conference on IT innovation. December 2004. Retrieved from <http://www.iir.hit-u.ac.jp/archive/event/WP05-08tanaka.pdf>

Tang, J. H., & Fam, C. K. (2005). The effect of interpersonal influence on softlifting intention and behaviour. *Journal of Business Ethics, 56*(2), 149-161. doi: 10.1007/s10551-004-2170-9

Taylor, S. A. (2012). Evaluating digital piracy intentions on behaviors. *Journal of Services Marketing, 26*(6-7), 472-483. doi: 10.1108/08876041211266404

Taylor, S. A., Ishida, C., & Wallace, D. W. (2009). Intention to engage in digital piracy a conceptual model and empirical test. *Journal of Service Research, 11*(3), 246-262. doi: 10.1177/1094670508328924

Tepper, S. J., & Hargittai, E. (2009). Pathways to music exploration in a digital age. *Poetics, 37*(3), 227-249. doi: 10.1016/j.poetic.2009.03.003

Tzantzara, K., & Economides, A. A. (2010). Gender differences in digital music distribution methods. *Peer-to-Peer Networking and Applications, 3*(2), 161-171. doi: 10.1007/s12083-009-0056-4

van Kranenburg, H., & Hogenbirk, A. (2005). Multimedia, entertainment, and business software copyright piracy: A cross-national study. *Journal of Media Economics, 18*(2), 109-129. doi: 10.1207/s15327736me1802_3

Waldfogel, J. (2010). Music file sharing and sales displacement in the iTunes era. *Information Economics and Policy, 22*(4), 306-314. doi: 10.1016/j.infoecopol.2010.02.002

Walls, W. D. (2008). Cross-country analysis of movie piracy. *Applied Economics, 40*(5), 625-632. doi: 10.1080/13504850600707337

Wang, C.-c., Chen, C.-t., Yang, S.-c., & Farn, C.-k. (2009). Pirate or buy? The moderating effect of idolatry. *Journal of Business Ethics, 90*(1), 81-93. doi: 10.1007/s10551-009-0027-y

Wang, J., Yang, Z., & Bhattacharjee, S. (2011). Same coin, different sides: Differential impact of social learning on two facets of music piracy. *Journal of Management Information Systems, 28*(3), 343-384. doi: 10.2753/mis0742-1222280310

Wang, X., & McClung, S. R. (2011). Toward a detailed understanding of illegal digital downloading intentions: An extended theory of planned behavior approach. *New Media & Society, 13*(4), 663-677. doi: 10.1177/1461444810378225

Wang, X., & McClung, S. R. (2012). The immorality of illegal downloading: The role of anticipated guilt and general emotions. *Computers in Human Behavior, 28*(1), 153-159. doi: 10.1016/j.chb.2011.08.021

Wingrove, T., Korpas, A. L., & Weisz, V. (2011). Why were millions of people not obeying the law? Motivational influences on non-compliance with the law in the case of music piracy. *Psychology Crime & Law, 17*(3), 261-276. doi: 10.1080/10683160903179526

Wolfe, S. E., Higgins, G. E., & Marcum, C. D. (2008). Deterrence and digital piracy: A preliminary examination of the role of viruses. *Social Science Computer Review, 26*(3), 317-333. doi: 10.1177/0894439307309465

Won, S. J., & Jang, J. (2012). Nonlinear income inequality effect on software piracy. *The Korean Journal of Economics, 19*(2), 213-242. doi: 10.2139/ssrn.1478907

Xia, M., Duan, W., Huang, Y., & Whinston, A. B. (2006). *Unravel the drivers of online sharing communities: An empirical investigation*. University of Illinois at Urbana-Champaign. College of Business, Working Papers. Retrieved from <http://www.business.uiuc.edu/Working_Papers/papers/06-0109.pdf>

Xia, M., Huang, Y., Duan, W., & Whinston, A. B. (2012). To continue sharing or not to continue sharing? An empirical analysis of user decision in peer-to-peer sharing networks. *Information Systems Research, 23*(1), 247-259. doi: 10.1287/isre.1100.0344

Yang, D., Sonmez, M., Bosworth, D., & Fryxell, G. (2009). Global software piracy: Searching for further explanations. *Journal of Business Ethics, 87*(2), 269-283. doi: 10.1007/s10551-008-9884-z

Yoon, C. (2011). Theory of Planned Behavior and ethics theory in digital piracy: An integrated model. *Journal of Business Ethics, 100*(3), 405-417. doi: 10.1007/s10551-010-0687-7

Yoon, C. (2012). Digital piracy intention: A comparison of theoretical models. *Behaviour & Information Technology, 31*(6), 565-576. doi: 10.1080/0144929x.2011.602424

Yu, S. (2010). Product placement and digital piracy: How young Chinese viewers react to the unconventional method of corporate cultural globalization. *Communication, Culture & Critique, 3*(3), 435-463. doi: 10.1111/j.1753-9137.2010.01079.x

Zamoon, S., & Curley, S. (2008). Ripped from the headlines: What can the popular press teach us about software piracy? *Journal of Business Ethics, 83*(3), 515-533. doi: 10.1007/s10551-007-9636-5

Zentner, A. (2005). File sharing and international sales of copyrighted music: An empirical analysis with a panel of countries. *B.E. Journal of Economic Analysis and Policy: Topics in Economic Analysis and Policy, 5*(1), 1-15. doi: 10.2202/1538-0653.1452

Zentner, A. (2008). Online sales, Internet use, file sharing, and the decline of retail music specialty stores. *Information Economics and Policy, 20*(3), 288-300. doi: 10.1016/j.infoecopol.2008.06.006

Zentner, A. (2009). *Ten years of file sharing and its effect on international physical and digital music sales*. University of Texas at Dallas. 2009. Retrieved from <http://papers.ssrn.com/sol3/papers.cfm?abstract_id=1724444>

Zentner, A. (2010). *Measuring the impact of file sharing on the movie industry: An empirical analysis using a panel of countries*. University of Texas at Dallas. March 22, 2010. Retrieved from <http://papers.ssrn.com/sol3/papers.cfm?abstract_id=1792615>
